# Optimizing proton minibeam radiotherapy by interlacing and heterogeneous tumor dose on the basis of calculated clonogenic cell survival

**DOI:** 10.1038/s41598-021-81708-4

**Published:** 2021-02-11

**Authors:** Matthias Sammer, Stefanie Girst, Günther Dollinger

**Affiliations:** Institut für Angewandte Physik und Messtechnik (LRT2), Universität der Bundeswehr München, 85579 Neubiberg, Germany

**Keywords:** Medical research, Oncology, Radiotherapy

## Abstract

Proton minibeam radiotherapy (pMBRT) is a spatial fractionation method using sub-millimeter beams at center-to-center (ctc) distances of a few millimeters to widen the therapeutic index by reduction of side effects in normal tissues. Interlaced minibeams from two opposing or four orthogonal directions are calculated to minimize side effects. In particular, heterogeneous dose distributions applied to the tumor are investigated to evaluate optimized sparing capabilities of normal tissues at the close tumor surrounding. A 5 cm thick tumor is considered at 10 cm depth within a 25 cm thick water phantom. Pencil and planar minibeams are interlaced from two (opposing) directions as well as planar beams from four directions. An initial beam size of σ_0_ = 0.2 mm (standard deviation) is assumed in all cases. Tissue sparing potential is evaluated by calculating mean clonogenic cell survival using a linear-quadratic model on the calculated dose distributions. Interlacing proton minibeams for homogeneous irradiation of the tumor has only minor benefits for the mean clonogenic cell survival compared to unidirectional minibeam irradiation modes. Enhanced mean cell survival, however, is obtained when a heterogeneous dose distribution within the tumor is permitted. The benefits hold true even for an elevated mean tumor dose, which is necessary to avoid cold spots within the tumor in concerns of a prescribed dose. The heterogeneous irradiation of the tumor allows for larger ctc distances. Thus, a high mean cell survival of up to 47% is maintained even close to the tumor edges for single fraction doses in the tumor of at least 10 Gy. Similar benefits would result for heavy ion minibeams with the advantage of smaller minibeams in deep tissue potentially offering even increased tissue sparing. The enhanced mean clonogenic cell survival through large ctc distances for interlaced pMBRT with heterogeneous tumor dose distribution results in optimum tissue sparing potential. The calculations show the largest enhancement of the mean cell survival in normal tissue for high-dose fractions. Thus, hypo-fractionation or even single dose fractions become possible for tumor irradiation. A widened therapeutic index at big cost reductions is offered by interlaced proton or heavy ion minibeam therapy.

## Introduction

Around 17 million new cases of cancer worldwide have been reported in 2018^1^. About 50% of cancer patients benefit from treatment with ionizing radiation^2^. The goal of radiotherapy is to sterilize all tumor cells while affecting the normal tissue as little as possible. The high doses needed for tumor control can cause severe damage to the surrounding healthy tissue limiting the therapeutic possibilities. Complementary to the standard sparing methods of radiotherapy such as temporal fractionation or intensity modulation, spatial fractionation is under current investigation to reduce side effects although first ideas have already been presented in 1909^3^ as “grid therapy” using several millimeter to centimeter beam sizes^4,5^, in particular for treating advanced bulky tumors.

The two main techniques considered for spatial fractionation with sub-millimeter beams are x-ray micro- and minibeam, as well as proton or heavy ion minibeam therapy. X-ray microbeam therapy (MRT) utilizing beams with sizes smaller 100 µm was introduced at the Brookhaven National Laboratory (New York, NY, USA)^6^ and further developed at the European Synchrotron Radiation Facility^7^. Heavy ion minibeam therapy was introduced by Dilmanian et al.^8^ and extended to unidirectional proton minibeam radiotherapy by Zlobinskaya et al.^9^ and Prezado et al.^10^. Dimensions of pencil or planar minibeams range between 0.1 and 1 mm with center-to-center (hereafter: ctc) distances of a few millimeters in proton (pMBRT) and x-ray minibeam radiation therapy (MBRT). The basic idea of x-ray and ion minibeams is the geometrical sparing of healthy tissue such that small parts of the volume receive very high doses (up to several hundred Gy) but large parts of the irradiation volume receive very low to no doses. Reduction of side effects in normal tissue has been experimentally verified in healthy mouse ears^11^ and rat brains^12^ with an observed dependency on the ratio of beam size σ (given by the standard deviation of the resulting dose profile) and ctc distances i.e. σ/ctc^13^. The resulting higher tissue tolerances are based on various kinds of the dose-volume effect^14,15^, which describes the volume dependency of the dose that induces a certain effect^16^, and the so-called “prompt microscopic biological repair effect” of blood vessels^17^. The mechanism behind the dose-volume effect, where organ damage increases with the irradiated (partial) organ volume, is assumed to be migration and repopulation capacities of irradiated areas by nearby healthy cells^18^. The repair potential is attributed in first order to the mean clonogenic cell survival while the size of individual minibeams gives upper limits where repair of the organ is effective^19,20^.

The beam size of proton and heavy ion minibeams increases with depth due to multiple small angle scattering. The dose modulation within the tumor is described by the σ/ctc ratio. Using a Gaussian as first order approximation for the lateral beam distribution from small angle scattering σ_sc_ as well as the initial beam size σ_0_, the beam size

1 $$\upsigma =\sqrt{{\upsigma }_{0}^{2}+{\upsigma }_{\mathrm{sc}}^{2}\left(\mathrm{d},\mathrm{E}\right)}$$

is given by the initial beam size σ_0_ and the width induced by small angle scattering σ_sc_(d,E), which is dependent on tissue depth d, kinetic energy E and the ion sort of the incoming particles. In principle, beam divergence has to be additionally considered widening the minibeam. In general, beam divergence of a focused beam contributes much less than small angle scattering [Mayerhofer et al. (in press), Medical Physics].

Particle energy, which determines the range, is adjusted to the tumor location. It is varied to form the so-called spread out Bragg peak (SOBP) that covers the tumor thickness with a uniform dose in depth. The mean dose is adjusted to obtain a high probability of tumor control at an acceptable level of side effects. The lateral dose modulation within the tumor is controlled by the ctc distances considering the lateral spread of the beam at a certain depth. Varying the ctc enables several possibilities for tumor irradiation. Homogeneous irradiation of the tumor according to the ICRU reports 50 and 62^21,22^ needs to fulfill σ/ctc > 0.5 for Gaussian beams^23^. By increasing the ctc distances, the σ/ctc ratio drops and the tumor receives a heterogeneous irradiation. To avoid cold spots within the heterogeneously irradiated tumor volume, the mean dose may be elevated such that a minimum required dose for tumor control is obtained even in the dose valleys. It has already been proven by Rivera et al. that the valley dose is the most relevant parameter for tumor control^24^.

**Figure 1:**
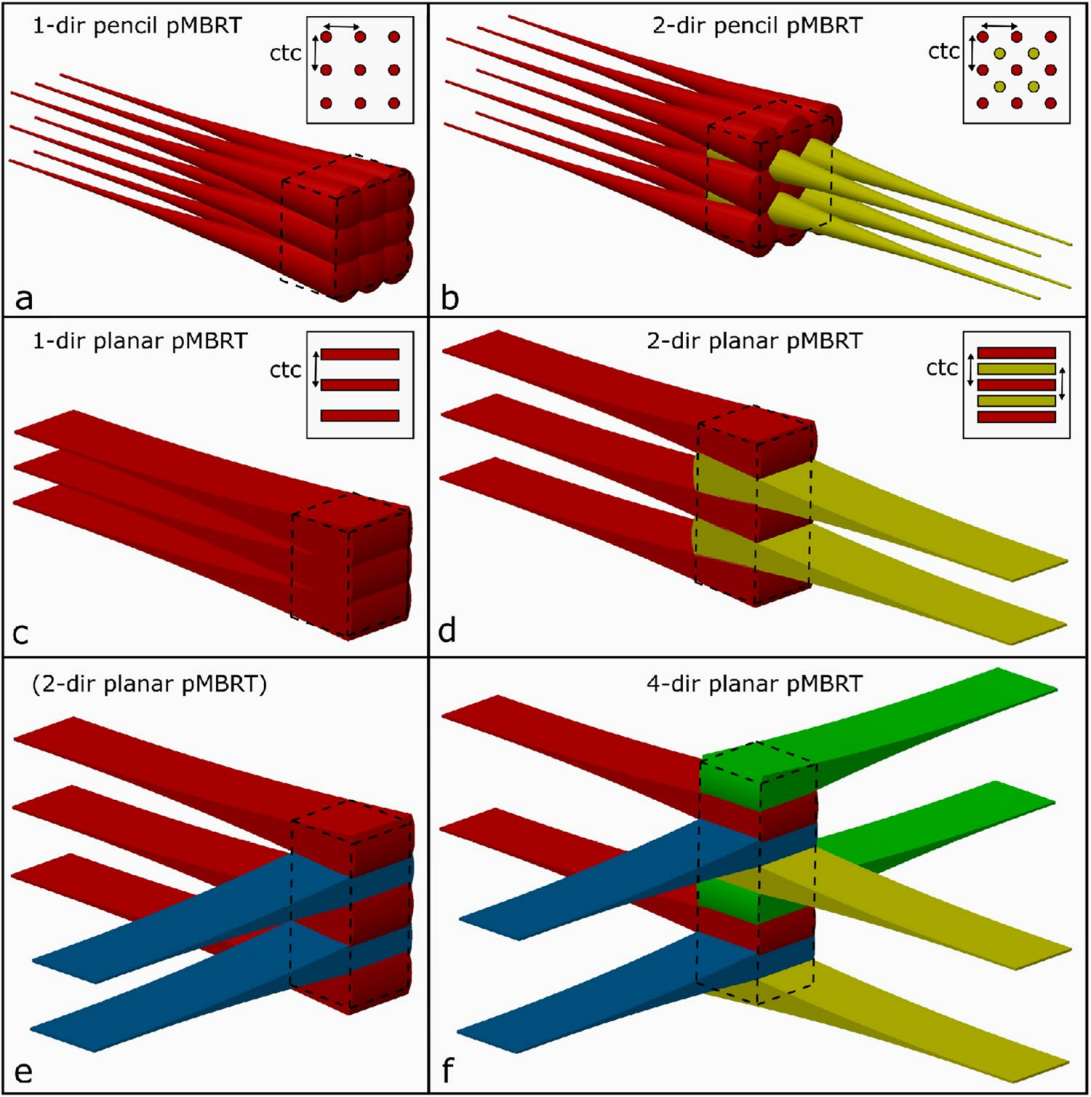
Schematic representation of unidirectional proton minibeams and interlaced minibeams from multiple directions. Unidirectional minibeams can be applied with pencil (**a**) or planar (**c**) minibeams. Pencil minibeams are interlaced from two opposite directions (**b**) with the two grids shifted by (ctc/2, ctc/2). Planar minibeams are interlaced from two opposing (**d**) and two orthogonal (**e**) directions with the two grids shifted by (ctc/2). Planar minibeams are also interlaced from four directions (**f**; 90° between all neighboring directions, grids shifted by ctc/4 between neighboring directions). Figures are created using the Creo 2.0 software. Credit: Peter Hartung.

Concerning unidirectional (1-dir) minibeam radiotherapy with protons (see Fig. 1a,c) (hereafter: pMBRT), dose distributions were already assessed by calculating the mean clonogenic cell survival^23^. A 5 cm thick tumor with its proximal edge at 10 cm depth was chosen as a model to be irradiated with protons in either broadbeam configuration or in spatially fractionated minibeams of various shapes. The tumor dose in the former study was kept homogeneous according to the ICRU reports^21,22^. Large sparing of healthy tissue due to strongly enhanced mean cell survival compared to homogeneous irradiation is achieved in the first centimeters below the skin. However, relative tissue sparing decreases with depth due to the small angle scattering of the minibeams. After about 7 cm of depth, cell survival is not substantially enhanced for proton minibeams compared to conventional proton irradiation. Hence, the close vicinity of the tumor does not benefit from spatial fractionation by 1-dir proton minibeam irradiation when a homogeneous tumor dose is considered. Besides, tissues in the close tumor surrounding are particularly critical since they receive similarly high doses as the tumor.

In order to improve tissue sparing towards the close surroundings of the tumor, σ/ctc ratios need to be decreased by reducing beam sizes or increasing ctc distances. Beam diameters depend on initial beam size and depth in tissue and are thus not easily changed for a given tumor scenario. In this work, the options for increasing ctc distances by interlacing (also interleaving, cross-firing) beams from two or more directions are studied. The minibeam arrays of several directions are adjusted such that the dose maxima of the minibeams from one direction are positioned in the dose minima of the minibeams from the other direction^8,25,26^. Pencil and planar minibeams are interlaced from two opposing directions (2-dir, Fig. 1b,d), leading to substantial tissue sparing only for particles due to their finite range, which is not the case for photons. As an additional possibility, planar beams are interlaced from four directions (4-dir, here with 90° angles between neighboring directions) (Fig. 1f). The neighboring directions are not necessarily orthogonal but angles < 90° lead to overlap in the healthy tissue in front of the tumor. This is the first time a real interlacing from 4 directions is presented, where every fourth minibeam within the tumor comes from one side. Suggestions to interlace from four directions was already described by Dilmanian et al.^8^ or Henry et al.^25,27^, but both approaches superpose two directions and only interlace the other two directions reducing the interlacing potential to that of two directional interlacing (shown for comparison in Supplementary Material Figure S1, Figure S2). For the sake of completeness, the interlacing of two orthogonal directions is also presented (Fig. 1e) but not considered in the actual dose calculation because of its similarity to the interlacing of two opposing directions.

For all cases, heterogeneous tumor irradiations are compared to homogeneous tumor doses to show their potential for enhanced sparing of the healthy tissue at the tumor edge by spatial fractionation. The upper dose constraint for prescribed doses as given by the ICRU reports^21,22^ is waived but the low dose limit within the tumor^28^ is kept for sufficient treatment dose covering the whole tumor. All dose calculations are assessed by a calculated mean clonogenic cell survival in view of sparing normal tissue from side effects as in the previous study^23^.

Interlaced proton minibeam irradiations require much larger technical efforts to adjust the minibeams from different directions with the necessary precision in terms of positioning and beam directions relative to their dose modulations in the tissue. Moving organs (tumors) are also an issue for interlaced proton minibeam irradiations. Dependent on tumor size, depth inside the body and robustness of the dose planning against variations, submillimeter to even 0.1 mm beam adjustments may be required within the tumor. This technical effort will only be considered when a clear advantage of interlaced compared to unidirectional proton minibeam irradiation modes is obvious. The present study is intended to show whether and under which conditions substantial increase in tissue sparing potential is obtained from interlaced proton minibeams applied on an idealistic tumor. Technical solutions to obtain all beam requirements for interlaced proton minibeam irradiations may be established from the results presented here.

## Materials and methods

Similar to the previous in-silico study from 2017^23^, dose distributions are calculated in Matlab^29^. The dose distributions of proton minibeams are approximated by Gaussian distributions resulting from the lateral spread through small angle multiple scattering of the protons. Data for the lateral spread of protons in water are taken from the database LAP-CERR^30,31^, which is based on Monte-Carlo simulated dose distributions that were fitted by either a single Gaussian distribution or a sum of two Gaussian distributions. Minibeams are assumed to be produced by focusing through an ion lens excluding the generation of secondary particles from collimation. Divergence of the proton beams is also assumed to be less than the spread through multiple scattering of the beam within the body and is neglected for the dose simulations.

Planar minibeams and quadratically arranged round pencil minibeams are considered. For the planar case, 17 minibeams are calculated while 16 × 16 minibeams are calculated for the pencil beam arrangements. The resulting unit cell at the center of the pattern delivers the representative dose distribution for each pattern. The numerical accuracy of the centered unit cell is < 8 × 10^–6^ D_mean_, where D_mean_ is the mean dose. The broadbeam scenario is assumed as laterally homogeneous. The binning for the dose calculations is 0.01 mm × 0.01 mm for each pixel.

The considered scenario is a tumor with its proximal edge located in 10 cm depth and a thickness of 5 cm as used in^23^. For irradiation from several directions, the 5 cm thick tumor is located at the center of a symmetrical 25 cm thick phantom, i.e. at 10 cm depth from each direction.

A spread-out Bragg peak (SOBP) is assumed to homogeneously cover the tumor in the longitudinal direction. The required fractions of protons of different energies are taken from the previous study obtaining a dose fluctuation of $\left|\frac{{\mathrm{D}}_{\mathrm{SOBP}}}{{\mathrm{D}}_{0}}\right|<$ 1.0% within the target volume^23^. The same fractions of particle energies, but laterally rearranged, are used for the minibeam calculations. Hence, the SOBP curve is the depth dose distribution for the broadbeam irradiation and delivers the mean dose of the minibeam irradiation at each depth.

Lateral dose distributions approximated by a single Gaussian are taken from the database LAP-CERR^30,31^ for depths between 10 and 157 mm. To get a better representation of the lateral dose distribution in particular in the valleys between the minibeams close to the tumor edges, a two-Gaussian representation was chosen and also taken from the LAP-CERR database. The two-Gaussian representation for lateral dose distributions of protons delivers more appropriate data than the one-Gaussian model at depths larger 4 cm, where large-angle scattering contributions start to increase^32^. The initial beam size at the patient is assumed as a pure Gaussian with σ_0_ = 0.2 mm (standard deviation). Ctc distances are optimized dependent on minibeam shape, number of beam directions and dose heterogeneity in the tumor volume. The scattering outside the restricted tumor volume (5 cm) is neglected for the one-Gaussian approximation since it only has a minor influence on the overall dose distribution, but is integrated for the two-Gaussian approximation.

Every calculated dose map D(x,y) is converted into a calculated clonogenic cell survival (hereafter referred to only as cell survival for simplified reading) S(x,y) by the linear-quadratic model given as S(x,y) = exp(− αD(x,y) − βD^2^(x,y))^33^. The α and β values are taken from the previous study^23^ with α = 0.425 Gy^−1^ and β = 0.048 Gy^−2^, i.e. α/β = 8.9 Gy being a mean value of the PIDE database for human cells, including both tumor and healthy tissue cells. The linear-quadratic model has limitations for high doses (i.e. > 10 Gy). Experimental data have shown a rather constant decrease in the logarithmic displayed cell survival curves for high doses, whereas the LQ-model curve bends with increasing dose, overestimating the cell killing. The linear continuation is included in a number of different cell survival models such as the Kavanagh–Newman^34^, Two components^35^, Linear-Quadratic Linear model^36^ and others^37–42^. The linear continuation for high doses can, however, not be generalized since the exact cell survival curve and also the appropriateness of a model depends on the cell type^43^. In addition, most advanced survival models include a further parameter, which is often unavailable in a database like PIDE. In spatial fractionation, where dose modulations need to be translated into a cell survival, an advanced model like the LQL-model might be slightly more accurate when describing the absolute clonogenic cell survival, but e.g. the maximum differences between the LQ-model and the LQL-model and their mean cell survival was found to be of < 0.04 percentage points in our previous minibeam study^23^. These small differences are based on the overall small cell survival results (for most cells ≪ 1%) found for doses higher than 10 Gy. The peak doses in proton minibeam therapy can be up to several hundred Gy resulting in a minor contribution to cell survival independent on the used model. Calculating the mean cell survival within a unit cell on a percentage scale is therefore independent of the accuracy of the model within the high dose regions. Increased clonogenic cell survival results mainly from the low doses (< 10 Gy) in the valleys where the cell survival is high on a percentage scale and accurately described by the LQ-model as also proposed by Guardiola et al.^44^. It needs to be mentioned, that the calculated clonogenic cell survival based on the LQ-model is used as a simple biologically weighted measure for spatially fractionated dose distributions, but precise biological consequences cannot be withdrawn from the data (more details are found in the discussion).

Non-targeted effects were not included in the calculations since they are diversely discussed^45–47^ and commonly accepted models to describe changes of cell survival due to lateral dose modulations are missing. In addition, they were attributed to “play a negligible role for low LET particles”^46,47^.

Dose dependent and energy dependent RBE of protons is not included in our calculations. RBE enhancements are reported to occur in dose valleys for collimated beams^48^. Similar enhancements, however, are not yet reported for focused beams where a much lower portion of protons is stopped in front of the Bragg peak. Thus, lateral changes in the RBE are not included in the calculations presented here.

The clonogenic cell survival for 2, 10 and 35 Gy homogeneous tumor dose is calculated for each irradiation scenario. In the case of heterogeneous dose distributions in the tumor, the same doses as assumed in the homogeneous case are set as minimum doses to be at least obtained at each location in the tumor. It should be noted that although high doses are assumed within the tumor, no absolute tumor control probabilities are modeled and all conclusions drawn for tumor control are only relative assessments.

## Results

### Irradiation with homogeneous tumor dose

**Figure 2:**
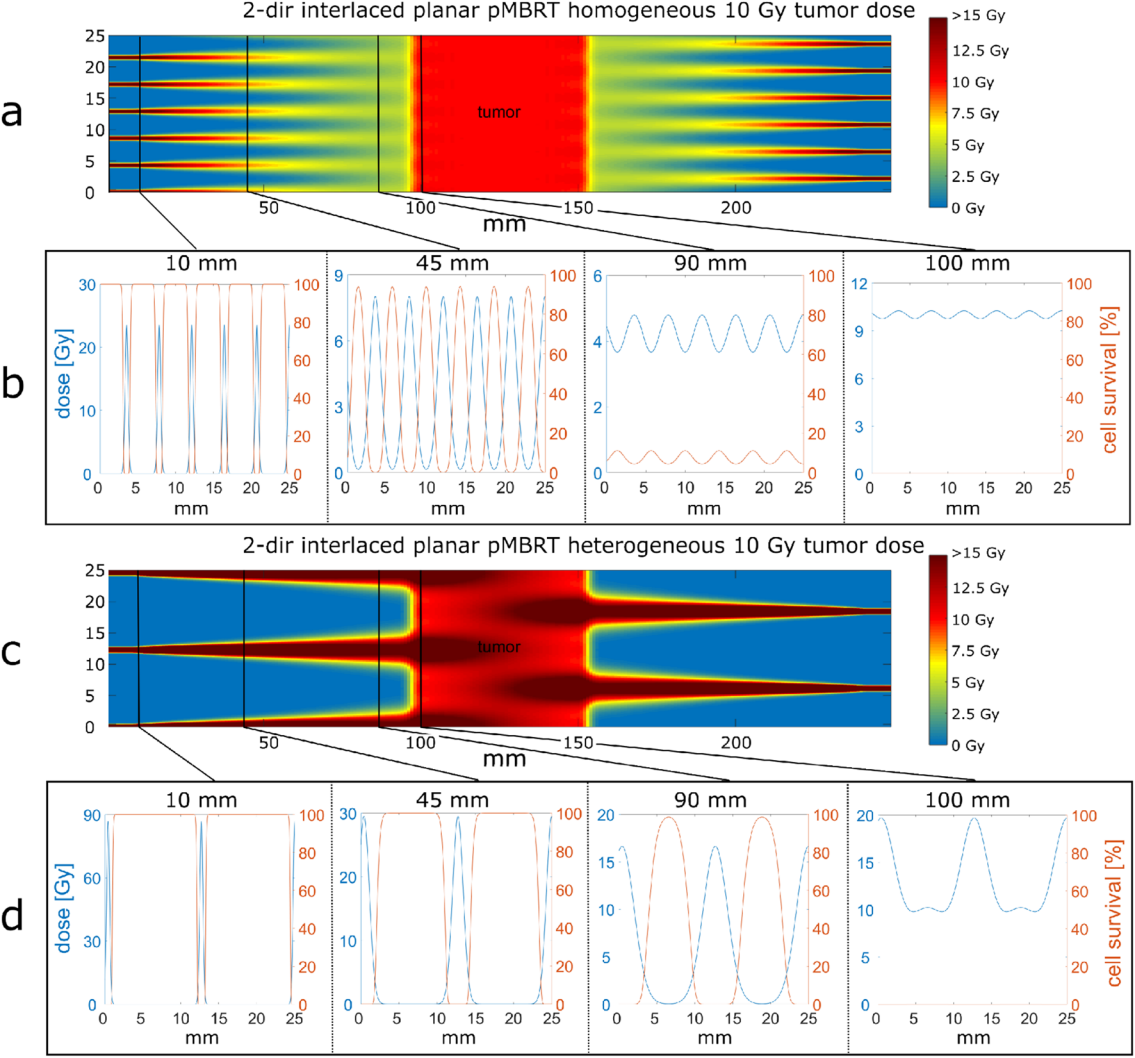
Dose maps of 2-dir interlaced planar minibeams (cf. Fig. 1c) with homogeneous (**a**) and heterogeneous (**c**) tumor dose. The dose is color-coded with saturation at 15 Gy. The cross-section of dose and the local clonogenic cell survival at 10, 45, 90 and 100 mm depth are shown for homogeneous (**b**) and heterogeneous (**d**) tumor irradiation with 10 Gy mean or minimum tumor dose, respectively. Heterogeneous tumor irradiation is described in section III.B.

Longitudinal cross-sections of dose distributions are plotted in Fig. 2a as an example for planar minibeams that are interlaced from two opposite directions (cf. Fig. 1d). The ctc distance between the minibeams is maximized under the constraint of homogeneous tumor dose: any location within the tumor phantom receives at least 97.5% but less than 103.5% of the prescribed tumor dose. These values are taken as a save range to fulfill the dosage criterion of the ICRU (95% < D_tumor_ < 107%) even under small uncertainties of dose calculations or applications. Figure 2a represents the dose profiles for a prescribed tumor dose of 10 Gy but can be linearly scaled to any other tumor dose.

One-dimensional cross-sections of the dose profiles (blue lines) and the corresponding local clonogenic cell survival (red lines) are plotted in Fig. 2b at depths marked in Fig. 2a. Close to the skin at 10 mm depth, the cell survival within the minibeams is negligible while in between, a plateau of nearly 100% cell survival is reached. The modulation of cell survival steadily decreases until a low oscillation and low overall cell survival is obtained close to the tumor.

**Figure 3:**
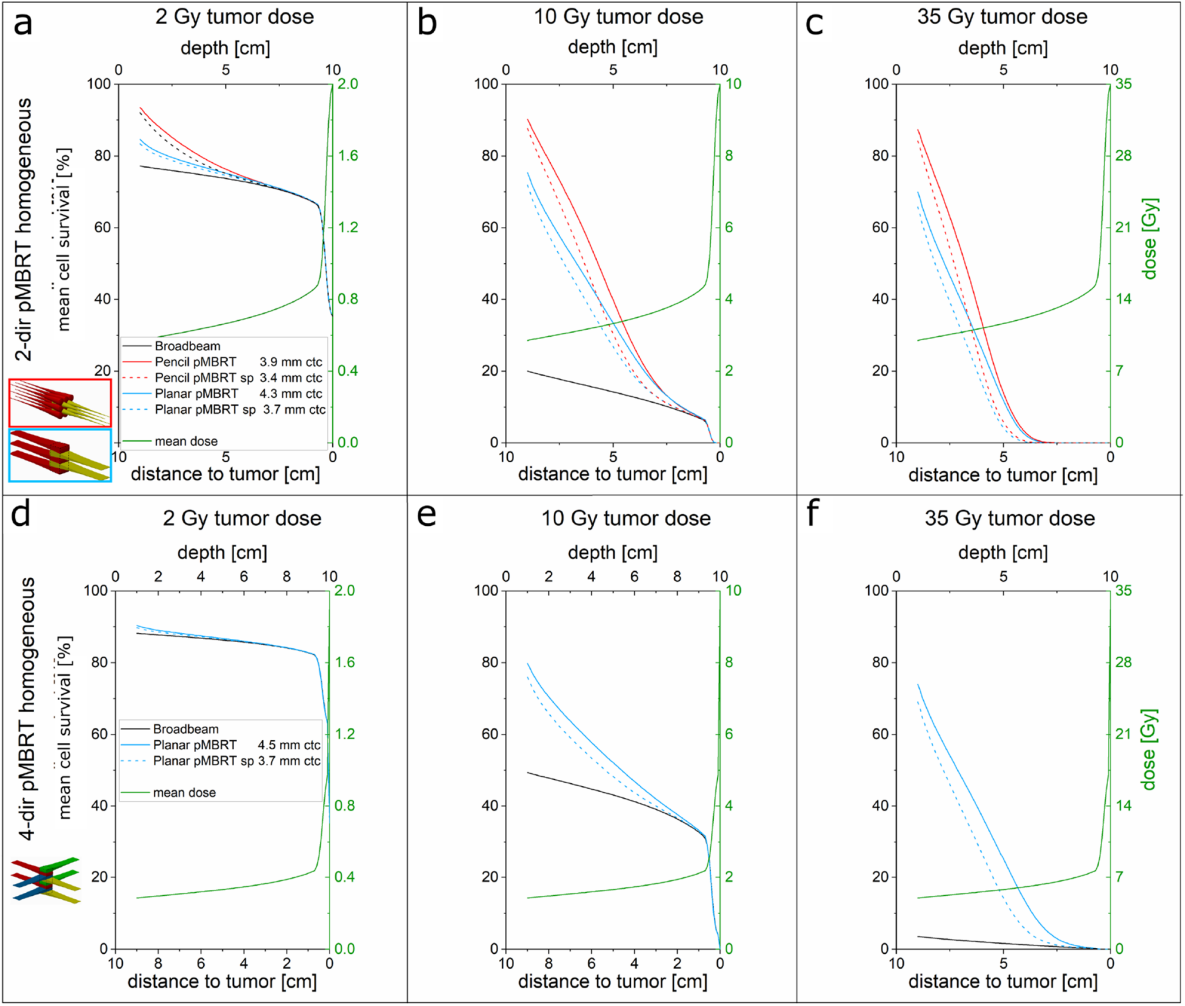
Mean cell survival for homogeneous tumor irradiation from two opposing directions (**a**–**c**) and four orthogonal directions (**d**–**f**). The tumor dose was set to 2 (**a**,**d**), 10 (**b**,**e**) or 35 Gy (**c**,**f**). “sp” is short for superposed and labels the irradiation by minibeams delivering a homogeneous tumor dose from each direction with half (2-dir) or quarter (4-dir) of the tumor dose delivered from each direction summing up to the prescribed tumor dose. The mean dose, which is the same for all irradiation modalities for homogeneous tumor irradiation, is added to each figure in green and corresponds to the y-axis on the right.

A major factor for the integrity and repair capacity of irradiated normal tissue is the mean cell survival within that tissue. The depth-dependent, clonogenic mean cell survival is calculated for comparison of the different prescribed homogeneous tumor doses (2, 10 and 35 Gy) for the various irradiation schemes of the 2-dir and 4-dir interlacing geometries in Fig. 3 (full lines). Due to symmetry, the cell survival curves along only one irradiated direction are plotted up to the proximal tumor edge. For comparison, mean cell survival for superposed 1-dir minibeam irradiations (dotted lines “sp”, Fig. 3) and for broadbeam irradiations (black lines) are calculated. Superposed 1-dir minibeam irradiations lead independently to the homogeneous irradiation of the tumor for each of the opposing or orthogonal directions (cf. Fig. 1a,c), with half or a quarter of the prescribed dose delivered from each direction for 2-dir or 4-dir, respectively. Summed up, the prescribed tumor doses are achieved as for the interlaced minibeam cases.

The cell survival curves for all minibeam irradiation cases show very high cell survival in the superficial layers. The interlaced pencil beams from two opposing directions (full red lines in Fig. 3) result in the highest cell survival of above 80% at 1 cm below the skin (9 cm in front of the tumor). Caused by the pure geometrical sparing, the cell survival in the entrance barely changes with the prescribed tumor dose. Even for a tumor dose of 35 Gy a mean cell survival greater 80% is obtained. With increasing depth, the cell survival curves of the minibeam irradiations approach the broadbeam survival levels (black lines in Fig. 3) until they eventually merge at 2 cm tumor distance. Hence, interlacing minibeams from multiple directions (full lines in Fig. 3) offers only a slight advantage compared to non-interlaced, superposed 1-dir minibeams (dotted line in Fig. 3), which are much easier to implement technically. The main cause is that the beams scatter within the tumor volume and the dose modulation decreases to the end of the irradiation range. At each tumor edge, the incoming beam has a high dose modulation, whereas the beam from the opposite direction that already passed the tumor has a lower dose modulation. The beam with lower dose modulation can hardly compensate for the high dose modulation of the beam entering the tumor. In combination with the strong homogeneity requirements within the tumor, ctc distances cannot substantially be increased compared to the unidirectional case.

The 2 Gy irradiations barely benefit from spatial fractionation since low doses have also high survival even for a broadbeam irradiation. For higher doses, the difference between the survival curves is more pronounced caused by the pure geometrical sparing from minibeam irradiation.

### Irradiation with heterogeneous tumor dose

To evaluate the tissue sparing potential by minibeam irradiation modes inducing a heterogeneous tumor irradiation, dose maps are calculated and the sparing potential is evaluated by the depth-dependent mean cell survival. An example of an optimized dose map for heterogeneous tumor irradiation by interlaced proton minibeams from two opposing directions is shown in Fig. 2c. The minimum dose within the tumor is set to 10 Gy, as it is the prescribed dose in the homogeneous case of Fig. 2a, but a dose modulation to larger doses is accepted. Thus, for heterogeneous dose profiles, the mean dose covering the tumor is increased by a factor f_D_ > 1 compared to the homogeneous case (f_D_ = 1) to obtain the same minimum dose as required. In Fig. 2c, the mean tumor dose is increased to 12.9 Gy, being a factor f_D _= 1.29 higher than the 10 Gy homogeneous dose of Fig. 2a. It results in dose maxima of 20 Gy when the ctc distance of the minibeams is maximized under these conditions to ctc = 12.3 mm. Thus, the ctc is 2.9 times larger than the ctc for the homogeneous case (ctc = 4.3 mm). The large ctc distances result in low dose levels within the dose valleys at all distances up to the close tumor surrounding. Thus, high local cell survival is maintained up to 10 mm in front of the tumor (Fig. 2d) with a high potential of reduced side effects.

**Figure 4:**
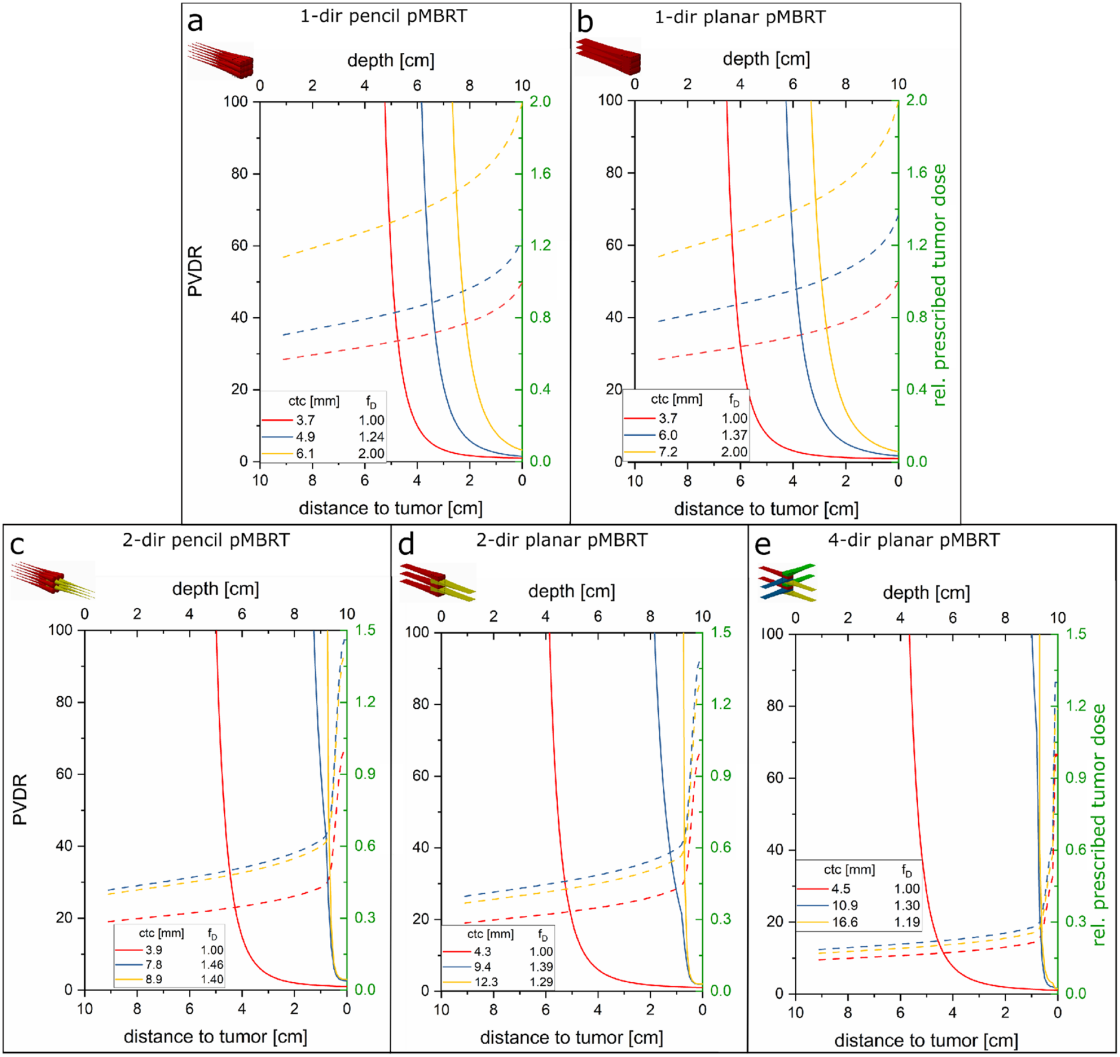
PVDR (full lines, left y-axis) and mean dose (dashed lines, right y-axis) for the heterogeneous (blue and yellow) vs. homogeneous (red) irradiation with planar (**a**) or pencil (**b**) unidirectional, two opposite (2-dir, **c** and **d**), and four-directional (4-dir, **e**) minibeams. The mean dose varies due to the dose modulation differences to fulfill the minimum dose constraint within the tumor. The required f_D_ for different ctcs’ are plotted in Fig. 6 as an example for 2-dir planar minibeams.

The depth-dependent peak-to-valley dose ratio (PVDR) and the mean dose is calculated for the various proton minibeam irradiation modes and plotted in Fig. 4. The mean doses are given relative to a prescribed minimum tumor dose.

The large PVDR > 100 at superficial layers (large distances to the tumor) is given by the assumed small initial beam size (σ_0_ = 0.2 mm) that will depend, however, on the technical conditions of a future proton minibeam irradiation facility. In any case, beam sizes and beam divergences are small at the superficial layers, beam sizes deeper in the body are mainly determined by the physical processes of small angle multiple scattering (see Eq. 1) as it is represented by the calculated PVDR in Fig. 4. The PVDR ratios only depend on the depth inside the body and the ctc distances. The ctc values can be adjusted to obtain large PVDR in particular at the close tumor vicinity but mean dose levels have to be raised by the factor f_D_ in order to fulfill the minimum dose criterion within the tumor.

**Figure 5:**
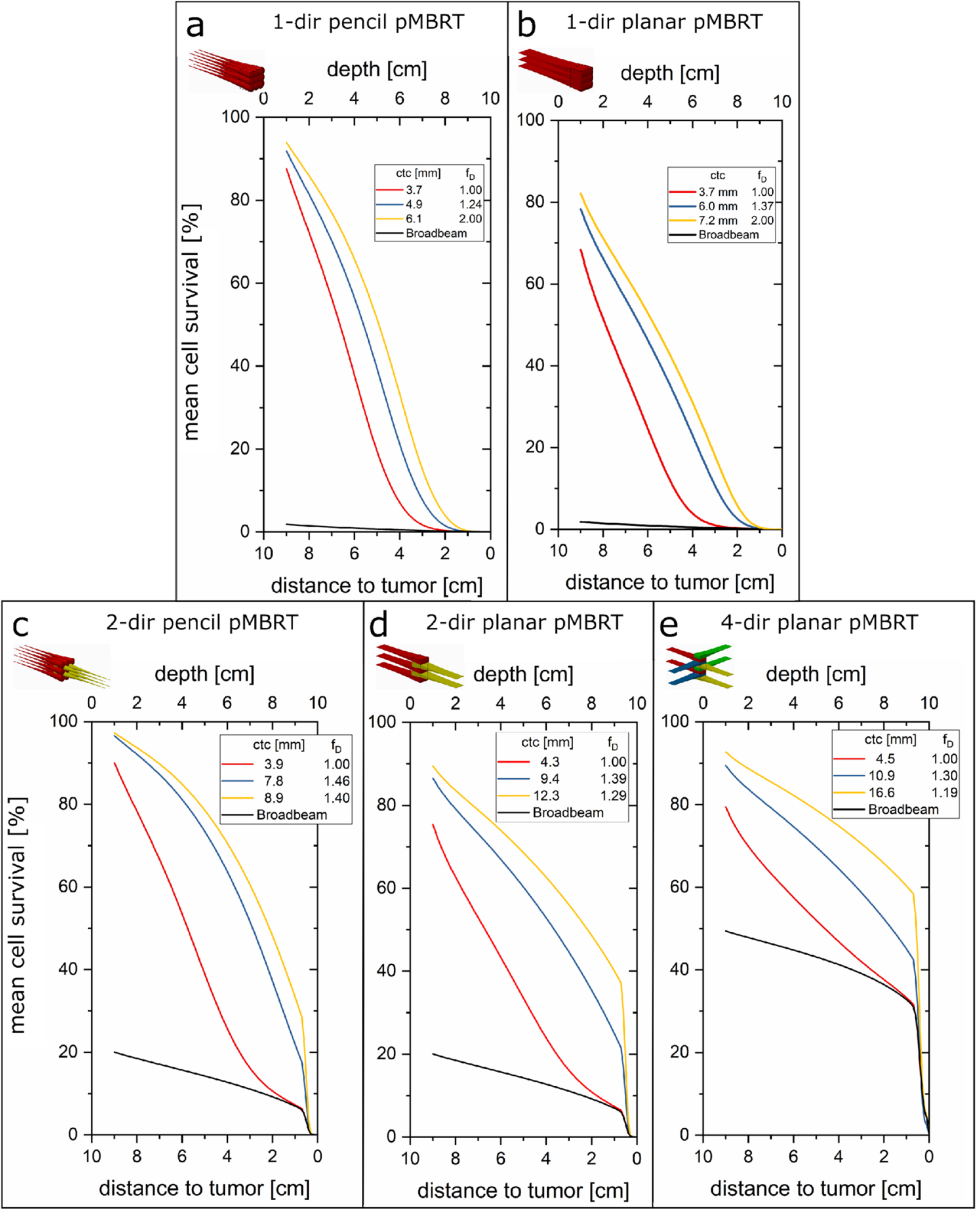
Mean cell survival results for the heterogeneous vs. homogeneous irradiation with planar (**a**) or pencil (**b**) minibeams from one direction, two opposite (2-dir, **c** and **d**) and four directions (4-dir, **e**). A minimum dose of 10 Gy within the tumor was applied.

For biological assessment, the depth-dependent mean cell survival is plotted in Fig. 5 for various irradiation scenarios of heterogeneous dose distributions in the 5 cm thick tumor and a minimum tumor dose constraint of 10 Gy. The mean cell survival of 1-dir pencil and planar proton minibeam irradiations is shown in Fig. 5a,b, respectively. The mean dose in the tumor was set to D_mean_ = f_D_ · 10 Gy as marked in the figures. Mean cell survival is larger for heterogeneous tumor irradiation of larger ctc distances but larger mean tumor doses (f_D_ > 1) compared to homogeneous irradiation (f_D_ = 1) already for the 1-dir case. Pencil minibeams result in larger mean cell survival than planar beams. However, cell survival strongly decreases with depth for the 1-dir cases and merges with a broadbeam irradiation scenario (black line in Fig. 5a, b) some centimeters before the tumor edge.

A strong increase in cell survival close to the tumor edge is achieved when 2-dir or 4-dir interlaced minibeams are applied with heterogeneous tumor irradiation. Mean cell survival for 2-dir, interlaced pencil and planar minibeam scenarios is presented for a 10 Gy minimum tumor dose in Fig. 5c,d. 4-dir interlaced (orthogonal) minibeam irradiation cases are plotted in Fig. 5e. Mean cell survival of the homogeneous cases (f_D_ = 1, red lines in Fig. 5) merge with the broadbeam irradiations (black lines in Fig. 5) 2–3 cm before the tumor edges. The heterogeneous cases show high levels of mean cell survival up to the close tumor vicinity. The steep slopes of the cell survival result from the distal sides of the SOBP arriving from the opposite direction at that tumor edge. Although the pencil minibeam arrangements lead to higher mean cell survival rates for homogeneous tumor irradiation in the 2-dir case, the interlaced minibeams with heterogeneous tumor dose show higher mean cell survival close to the tumor edge for planar minibeams. Altogether, heterogeneous dose distributions in the tumor are favored due to high mean cell survival within the healthy tissue in particular close to the tumor.

**Figure 6:**
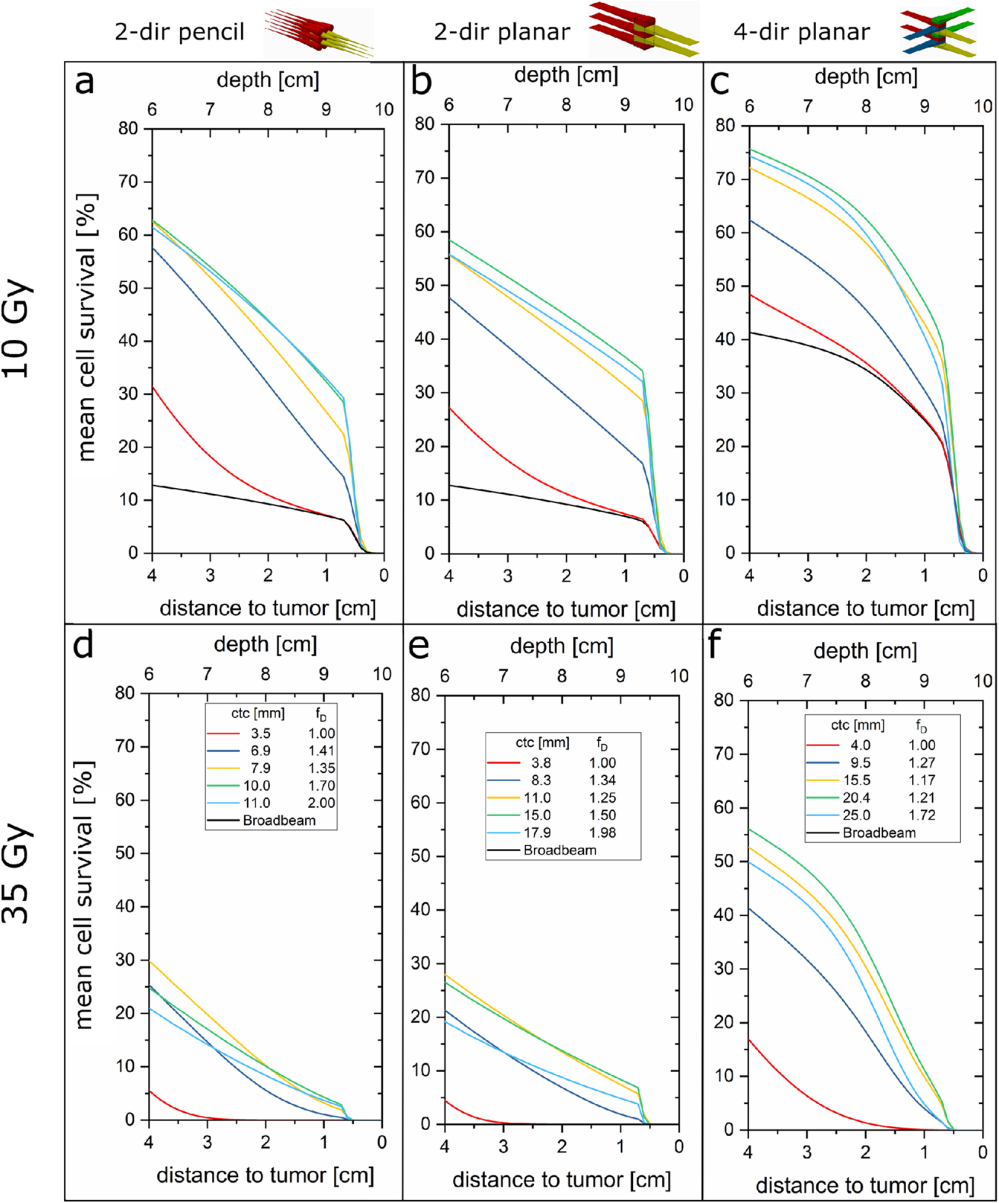
Mean cell survival results for the heterogeneous vs. homogeneous irradiation with 2-dir pencil (**a**,**d**) or planar (**b**,**e**) minibeams as well as 4-dir planar minibeams (**c**,**f**) at the close vicinity of the tumor using the two-Gaussian model from LAP-CERR^30,31^. The high doses of 10 Gy and 35 Gy minimum dose are presented in the upper row (**a**–**c**) and lower row (**d**–**f**), respectively. The cell survival for 35 Gy broadbeam irradiation is equal to 0 for all depths.

The simulations are extended using a more detailed representation of the lateral spread approximated by a two-Gaussian lateral dose distribution of the proton minibeams. It results in more reliable cell survival data for the dose valleys close to the tumor. The ctcs and the respective enhancement factors f_D_ were optimized for heterogeneous tumor irradiation modes and compared to broadbeam and homogeneous minibeam irradiation modes for 10 Gy and 35 Gy minimum tumor doses in Fig. 6. Optimized mean cell survival at the tumor edges are found from the calculated mean cell survival.

The resulting optimum ctc distances and mean cell survival levels are reported in Table 1.

**Table 1:** Comparison of different interlacing minibeam modes with 2-Gaussian approximation.

MB mode	ctc [mm]	f_D_	1 cm D_min_ = 10 Gy			9 cm D_min_ = 10 Gy			1 cm D_min_ = 35 Gy			9 cm D_min_ = 35 Gy	
			S [%]	d_eff,10%_[mm]	d_eff,50%_[mm]	S [%]	d_eff,10%_[mm]	d_eff,50%_[mm]	S [%]	d_eff,10%_[mm]	d_eff,50%_[mm]	S [%]	d_eff,10%_ [mm]
2-dir Broadbeam	–	1.00	20	–	–	7	–	–	~ 0	–	–	~ 0	–
4-dir Broadbeam	–	1.00	50	–	–	25	–	–	4	–	–	~ 0	–
2-dir pencil hom	3.5	1.00	87	1.1	1.3	7	–	–	84	1.3	1.5	~ 0	–
2-dir planar hom	3.8	1.00	73	0.7	0.9	7	–	–	67	0.9	1.1	~ 0	–
4-dir planar hom	4.0	1.00	78	0.7	0.9	25	–	–	71	0.9	1.1	~ 0	–
2-dir pencil hetero (opt)	10.0	1.70	98	1.4	1.5	32	6.7	8.6	97	1.6	1.7	4	9.8
2-dir planar hetero (opt)	15.0	1.50	91	1.0	1.1	37	5.7	7.7	90	1.3	1.4	8	8.5
4-dir planar hetero (opt)	20.4	1.21	94	0.9	1.1	47	4.7	7.1	93	1.1	1.3	11	8.1

For each minibeam mode, the homogeneous tumor irradiation and the optimum heterogeneous tumor irradiation is presented. The mean cell survival (S) and biologically effective beam size (d_eff_) for D_min_ = 10 and D_min_ = 35 Gy (minimum) tumor dose are given for 1 cm and 9 cm depth. The survival fractions of 10% or 50% are taken for determination of the effective beam sizes d_eff,10%_ and d_eff,50%_. Values marked by a dash (–) cannot be determined.

An optimum distance of ctc = 15 mm with f_D_ = 1.5 is obtained for 2-dir interlaced planar minibeams. A ctc = 20.4 mm with f_D_ = 1.21 is the optimum in the case of the four-directional proton minibeam irradiations. The high minimum tumor dose of D_min_ = 35 Gy leads to a drop in cell survival compared to the D_min_ = 10 Gy minimum tumor dose. However, there is still a substantial mean cell survival even at a high single dose fraction of 35 Gy.

**Figure 7:**
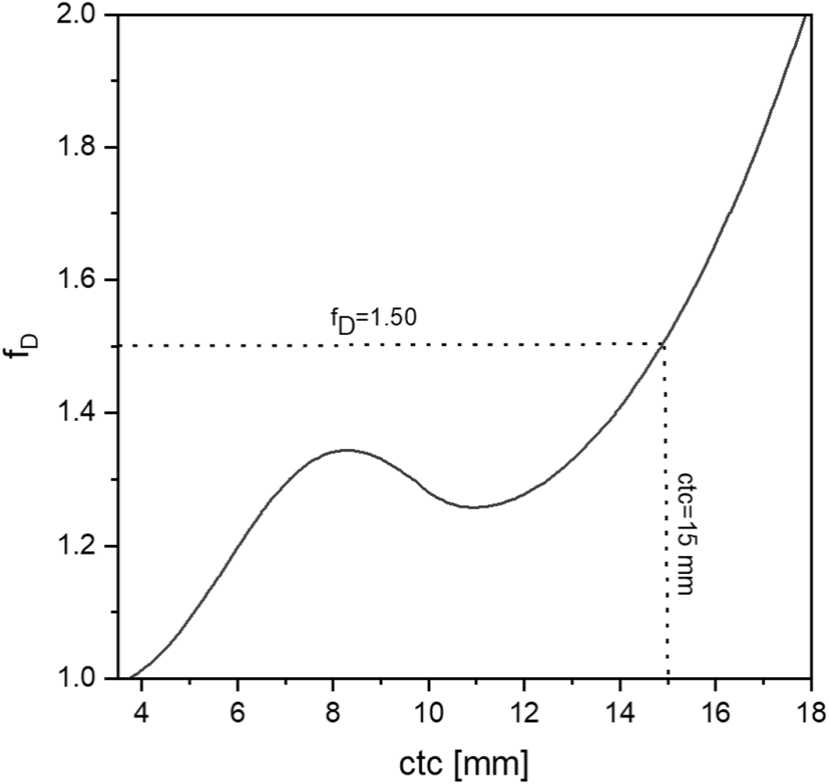
Multiplication factor f_D_ over ctc required to fulfill the minimum criterion D_min_/D_t_ = 0.975 for 2-dir interlaced planar minibeams. The beam shape was approximated with two Gaussians. The dotted lines mark the optimum found according to the calculated cell survival curves at the close tumor surrounding.

The minimum dose of the heterogeneous irradiation is set equal to the mean dose of conventional irradiation approaches since it mainly qualifies the tumor control^24^. This leads indispensably to an increase of the mean dose of heterogeneous irradiations by a factor f_D_ compared to the homogeneous irradiation. The dose enhancement factor f_D_ does not monotonically increase with ctc distances. For 2-dir interlaced planar minibeams and two-Gaussian approximation, it has a local maximum at ctc = 8.3 mm (f_D_ = 1.34) and a local minimum at ctc = 11 mm (f_D_ = 1.25) as shown in Fig. 7.

Although the mean cell survival slightly increases further beyond the minimum, a ctc close to the minimum seems to be a good option for a robust proton minibeam irradiation. The local minimum is the compromise between large ctc for tissue sparing but also high minimum dose without immense increase in the f_D_ factor. Ctc distances close to that minimum allow a robust tumor irradiation where minor variations of the ctc would barely change the dose coverage of the tumor.

### Biologically effective minibeam sizes

**Figure 8:**
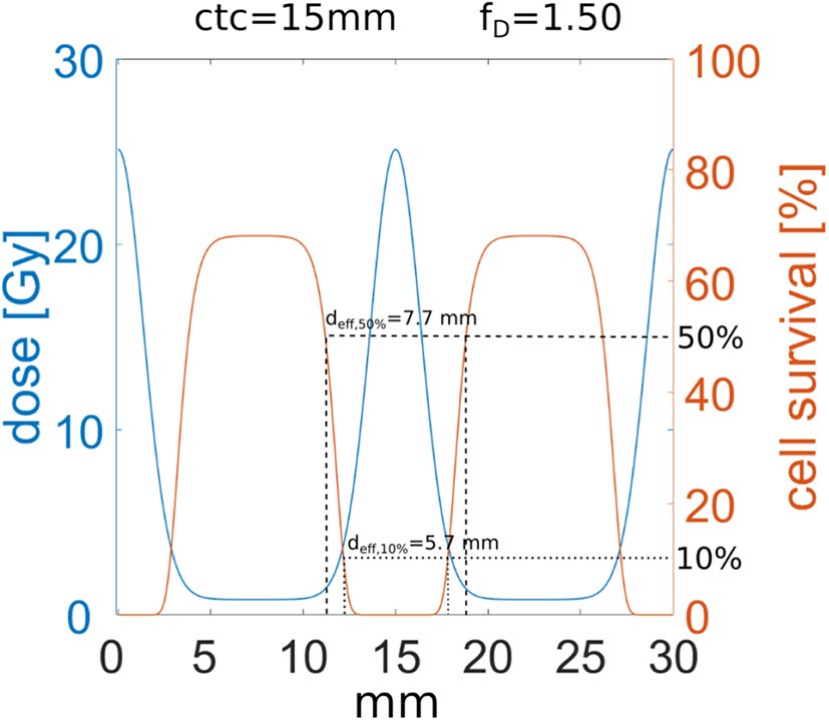
Dose profile (blue) and cell survival profile (orange) for 2-dir planar minibeams with ctc = 15 mm at 9 cm depth (2-Gaussian approximation). The dotted lines mark the biologically effective beam size with a 10% survival threshold (d_eff,10%_). The dashed lines mark the biologically effective beam size with a 50% survival threshold (d_eff,50%_). A ctc of 15 mm was found to deliver an optimum mean cell survival even though the mean dose has to be increased by the factor f_D_ = 1.50.

When irradiating deep lying tumors, initially small minibeams suffer from lateral spread through small angle scattering. The minimum beam size given by the standard deviation σ of the minibeams’ dose profile depends on the type of ions (e.g. protons, helium or carbon ions), the beam energy and the depth within the tissue. The lateral dose profile as obtained at 9 cm depth applying 2-dir planar proton minibeams with 15 mm ctc and f_D_ = 1.5 to the considered tumor model is shown in Fig. 8. The calculation is performed using the 2-Gaussian approach for the lateral dose profiles. To quantify the size of the minibeam, where most cells have radiation-induced damages, the biologically effective minibeam size d_eff_ is introduced. It depends on the lateral distribution of the absolute (physical) dose of an individual minibeam and, as the simplest approach, on a threshold fraction S_thres_ of proliferating cells, i.e. all cells within a circle of diameter d_eff_ have a survival probability ≤ S_thres_. As two examples, the effective beam sizes are shown for a threshold cell survival of S_thres_ = 50% (d_eff,50%_) and S_thres_ = 10% (d_eff,10%_) in Fig. 8 and presented in Table 1 for the various minibeam irradiation modes of the tumor model. The effective beam sizes usually only increase slightly with tumor dose. While effective beam sizes are well below 2 mm in diameter for any irradiation modality and dose of the considered tumor model in the superficial layers (≤ 10 mm depth), up to 10 mm effective beam sizes are obtained close to the tumor at 90 mm depth. A general presentation of the biologically effective minibeam sizes in dependence of maximum dose of a Gaussian minibeam and for different assumed threshold fractions S_thres_ of proliferation are presented in Figure S5 of the supporting material.

## Discussion

Spatial fractionation can substantially reduce side effects when the dose is distributed into small planar or pencil channels within the healthy tissue. The clonogenic mean cell survival is taken as a first order biological measure to compare different minibeam irradiation scenarios with regard to their tissue sparing potential. The higher the mean cell survival the lower the expected side effects. In total, the mean cell survival is mainly determined by the σ/ctc ratio and to a minor extent to the mean dose of the minibeam irradiated area. This holds true as long as the PVDR is not limited by beam halos. The utilized linear-quadratic model for calculating the clonogenic cell survival is inaccurate at high doses occurring in the minibeam peaks and could thus be replaced by more complicated cell survival models like the LQL model, as discussed in the “Materials and Methods” section. However, on the percentage scale of the calculated mean cell survival, the effect is negligible.

A second main ingredient, the effective beam size, influences sparing potential in spatial fractionation schemes as demonstrated in the mouse ear experiments of Sammer et al.^13,20^. It remains unclear how effective beam sizes influence tissue sparing in minibeam irradiation in other organs and in human tissue and will be discussed more at the end of this chapter. Therefore, the optimization of minibeam irradiation modes is based on mean cell survival comparisons only.

It needs to be mentioned here, that the calculations made are neither conducted with regards to specific tissues nor to specified side effects. Within a whole organism, side effects are also affected by several other mechanisms like the integrity and function of cells that do not proliferate at all or do no longer proliferate after irradiation, immune responses^49^ or bystander effects^50^. The mean clonogenic cell survival serves therefore only as a measure that weighs dose distribution with a biological background, but cannot forecast a whole radiation response. Different tissue types might not react according to a mean clonogenic cell survival if hot spots hit particularly sensitive tissues (e.g. serial tissues as spinal cord). Also, similar mean cell survival for different tissues might also have a different severity in radiation response. For tumor tissues it is not clear yet if a minimum dose is the only parameter of focus for tumor control. Hot spots as they appear from heterogeneous irradiation might be beneficial for tumor control by inducing immune responses^51^. Further investigations for healthy tissues and tumors need to be elaborated to gain a basic understanding of effects from valley, peak and even mean doses. A development of more sophisticated tissue models might also be important to translate spatially fractionated dose distributions into normal tissue complication probabilities (NTCP) and tumor control probabilities (TCP).

In our approach, we assumed a beam preparation by ion beam focusing. This is an attractive method, since lots of secondary radiation from collimation is prevented. However, it is technically more challenging even though there are already first attempts for pre-clinical designs [Mayerhofer et al. (in press), Medical physics] and even for a clinical accelerator^52^. Using a collimator for beam preparation, beam halos from additional scattering effects decrease peak-to-valley dose ratios compared to focused beams^52^. The halos depend on the details of collimator material, its geometry, the beam parameters and the collimator-beam adjustment.

Additional RBE effects from the scattered particles^48,53^ also reduce the sparing within the dose valleys, but probably play a less important role when a focused beam is chosen.

In the following discussion, we often refer to lower side effects and enhanced tissue sparing based on our results from mean clonogenic cell survival calculations as a first order approach, but the limitations of this approach need to be kept in mind.

### Heterogeneous versus homogeneous tumor irradiation

In conventional external beam radiotherapy, the homogeneity dose criteria within a tumor are required by international standards^21,22^. The principle of radiotherapy assumes that a tumor will be eliminated if any tumor stem cell is stopped from proliferation. Thus, a minimum dose is required at any position in the tumor. On the other hand, the normal tissue needs to be spared as much as possible such that no severe consequences for the patient occur. The upper dose limit as proposed e.g. by the ICRU report^21,22^ is mainly based on minimizing side effects in normal tissue rather than any adverse tumor control effects^54^. If the constraint on the upper dose level counteracts healthy tissue sparing it can be waived when larger dose fluctuations within the tumor can lead to lower side effects^28,55^.

The calculated mean clonogenic cell survival can be enhanced when proton minibeams are applied from two or more sides by interlacing the minibeam arrays (Figs. 5, 6) and thus increasing the ctc distance. Interlacing implies that the dose maxima of the minibeams from one side are placed at the dose minima of the minibeams from the other side(s) (Fig. 1). However, since the widths of the minibeams from various directions differ in particular at the tumor edges, the ctc cannot be much enlarged when the homogeneity criteria of the ICRU report^21,22^ need to be fulfilled. Thus, tissue sparing of interlaced minibeams is not much improved compared to superposed 1-dir minibeam applications where homogeneous dose distributions are obtained for each of the irradiation directions. In particular, mean cell survival and thus tissue sparing effects close to the tumor edges remain low in both cases (Figs. 5, 6, see also Table 1).

Mean clonogenic cell survival is enhanced in the healthy tissue even close to the tumor edges by interlacing proton minibeams when a heterogeneous dose distribution in the tumor is accepted. The only requirement is a minimum dose at any location in the tumor to achieve a tumor control at least as low as for the same homogeneous dose. For heterogeneous tumor irradiation with minimum dose D_min_ a higher mean dose is obtained, enlarged by a factor f_D_ > 1 compared to a homogeneous tumor irradiation with dose D_min_. In the presented study, D_min_ = 10 Gy was set to be compared with the same homogeneous dose in the tumor, but also D_min_ = 2 Gy and D_min_ = 35 Gy were considered. Mean clonogenic cell survival of heterogeneous proton minibeam irradiation modes is enhanced compared to that of the corresponding homogeneous irradiation modes in healthy tissue. The gain in cell survival in the dose valleys within the healthy tissue by enlarged ctc distances outnumbers its loss caused by the higher mean dose that has to be applied to the tumor to fulfill the minimum dose requirement. The resulting mean cell survival as obtained in superficial layers as well as 1 cm in front of the tumor edge is presented in Table 1. It demonstrates in detail the large enhancement in mean clonogenic cell survival compared to broadbeam, 1-dir minibeam, 2-dir or 4-dir minibeam irradiations with homogeneous dose distributions in particular close to the tumor edges. Thus, lower side effects can be expected close to the tumor edges compared to any other proton irradiation mode with the same number of beam directions. Within the tumor, the higher mean tumor dose results in even enhanced tumor control in combination with the already advantageous heterogeneous tumor irradiations according to Prezado et al.^56^. The advantages of the heterogeneous tumor irradiation could diminish the necessity of the same minimum dose as in homogeneous tumor irradiation but needs further experimental clarifications.

Comparing the 2-dir irradiations with interlaced pencil and planar minibeams, large differences in the applicable ctc distances can be noticed. While interlaced pencil minibeams of ctc = 11 mm require a doubling of the mean dose (f_D_ = 2), planar minibeams allow for 17.9 mm ctc for the same increase of the mean dose. The larger possible ctc distances for the planar case diminish the geometrical advantages of the pencil beams in the entrance channel. While between 90 and 98% mean clonogenic cell survival can be found for interlaced pencil minibeams in the superficial layers, the planar minibeams show only 75–95% (D_min_ = 10 Gy). However, at the tumor edges, higher mean cell survival with up to 37% can be found for planar minibeams in comparison to up to 32% for pencil minibeams (D_min_ = 10 Gy). Another advantage of planar minibeams is that beam production as well as beam delivery and positioning may be technically simpler than for pencil minibeams. Focusing strength of single magnetic quadrupole lenses to form a planar focus is much higher than the duplet arrangement of quadrupoles necessary for pencil minibeams. Besides, high precision to interlace planar beams is only required in one dimension. However, it remains to be investigated by radiobiological experiments whether spatial fractionation by pencil minibeams is more effective in tissue sparing compared to planar minibeam arrangements even in cases where mean cell survival is somewhat smaller than in the planar case.

From a geometrical point of view, we found that for centered tumors the most favorable interlacing geometry is irradiation with planar minibeams from four directions due to the quartering of the dose and the large possible ctc distances from each direction. The optimum modelled case allows for a ctc distance of 20.4 mm when the mean dose is f_D _= 1.21 times larger within the tumor. The optimized mean cell survival of 46.9% at 10 mm in front of the tumor is obtained with these parameters at a minimum tumor dose of D_min_ = 10 Gy. Nevertheless, it needs to be elucidated in future studies whether a cell survival of 47% from four directions is more beneficial than a 37% cell survival from two directions meaning that only half the volume of healthy tissue is affected. Using a single-energy distal edge approach for 2-dir opposing irradiation schemes instead of a spread-out Bragg peak might become a technically simplified approach for interlaced minibeams with heterogeneous tumor dose.

### Hypofractionated interlaced minibeam irradiation

Mean cell survival in the healthy tissue up to the tumor edge with the applied (heterogeneous) dose in the tumor can be held at significant levels (> 5–10%) even for D_min_ = 35 Gy dose minima in the tumor and mean doses of up to 70 Gy. Nevertheless, it will be important to translate and quantify cell survival into actual side effects as done in a first approach by Wheldon et al.^57^. Cell survival is not only dependent on dose but also on tissue type and will therefore cause clear effort in future quantification studies of tissue response to radiation.

The high cell survival up to the tumor edge allows for hypofractionation or even single fraction treatment. In addition, best tissue sparing by overlaid temporal fractionation of minibeam irradiation modes would require a well-adjusted reproduction of the irradiation arrays from one fraction to the next to obtain best overlap of the lateral dose profiles. It has been proven in a mouse ear model that a precise adjustment of four temporal daily fractions results in much better tissue sparing than when each of the temporal fractions was shifted by half a ctc distance^58^. Sub-millimeter positioning of the minibeams from one irradiation to the next requires large technical effort and might be hindered by organ movement. Hence, a single fraction might be the best option to use spatial fractionation instead of temporal fractionation for tumor irradiation. Intrafractional organ movement might be reduced by applying flash irradiation protocols to avoid dose inaccuracies. An option for a few temporal fractions without requiring the interfractional sub-millimeter position accuracy would be e.g. pairwise applied interlaced minibeam fields from several directions within the three dimensions. This approach is similar to the one of Serduc et al. for MRT but with the difference that a sufficient tumor coverage in interlaced MRT is achieved only after application of all MRT fractions or beam directions^26^. The advantage using ions is the pairwise interlacing that covers the whole tumor with a defined minimum dose in each fraction. The finite range of the ions allows several incident angles for pairwise interlacing. As a result, the irradiation directions between temporal fractions would only have a minor overlap in the healthy tissue.

### Biologically effective minibeam sizes

The tissue sparing potential by spatial fractionation depends on the kind of irradiated normal tissue, in particular, whether being parallel or serial. The survival and function of partially irradiated organs are described by the dose volume effect^14,15^. The injury of serial organs is dominated by the maximum appearing dose^59^. Hence, it will be important to be aware of the absolute positioning of the minibeams within these organs at risk. In contrary to organs at risk, the positioning of minibeams can also be used to efficiently target aggressive cancer cells within high-risk tumor subregions^60^.

In parallel organs, small lesions within the organ will not lead to a dis-function as long as parts of the organ remain intact. Examples for this behavior are liver, lung, and in part skin tissue when the lesions are small. The sizes of destructed lesions that are acceptable by the organs are in general not yet well known. As proven for skin tissue in a mouse ear model, the skin does not show much reactions when 0.18 mm channels are irradiated by high doses (~ 6000 Gy) leaving skin areas between the channels at very low doses^11^. An additional experiment showed that reactions are barely visible for irradiations with a single pencil beam of ≤ 2 mm diameter while a gradual increase of inflammation reactions is detectable with the diameter of a single beam increasing beyond 3 mm^20^. The inflammation in the same ear model also gradually intensifies for a grid irradiation of 16 pencil minibeams when the ratio of beam size to center-to-center distance σ/ctc increases much above 0.1^13^. Skin repair is associated with repopulation and migrating healthy, proliferating cells from low dosed areas to the heavily irradiated ones. Hence, the high dose regions will recover although the cells that were originally present in the high dose region will not proliferate any more with high probability. Characteristic migration lengths limit the size of damaged skin areas that are efficiently repaired.

As a consequence, radiation responses are not only influenced by mean cell survival but also by the effective minibeam sizes. It is necessary to evaluate biological responses of various organs to spatial fractionation in dependence of the effective minibeam sizes. The study in the mouse ear model suggested a major reduction of side effects for effective beam sizes below 3 mm^20^ although even larger beam sizes resulted in some reduction of radiation responses. The study was performed for pencil beams only. It remains to be clarified whether size limits differ between pencil and planar beams, which limits are given for different organs and how these limits change from small animals to human tissue.

In particular, it currently remains unclear which biologically effective beam sizes are acceptable between different species and tissues in order to profit most from spatial fractionation. For deep lying tumors, the effective proton minibeam sizes can be well above 3 mm as demonstrated for the 5 cm thick tumor at a depth of 10 cm as considered here (Table 1). Beam sizes become even wider when tumors are situated deeper in the body. Heavier ion beams like helium or carbon beams experience much less lateral spread and could be used to form smaller minibeams deep in the body if needed^8^. The lateral spread of helium beams is about half of that of proton beams, that of carbon beams about a quarter^32,61^. However, carbon ion therapy suffers from a considerable beam fragmentation tail at the distal end of the tumor. Thus, helium ions, that suffer much less from fragmentation, may be a good compromise for reduced effective beam sizes since they maintain low valley doses at the edges of deep lying tumors for interlaced minibeam treatment even at high minimum tumor doses as required for hypofractionation^61^.

Besides the fragmentation dose tail beyond the Bragg peak and the different RBE contributions for the heavier ions, interlaced minibeam geometries can be taken the same as for the proton case, but the ctc distances need to be decreased according to the reduced beam width σ keeping the σ/ctc ratio as in the proton case. Dose heterogeneity in the tumor and mean cell survival in the healthy tissue remains similar as calculated for protons but the lateral dimensions are smaller. Thus, spatial fractionation could profit from helium or carbon ions according to the reduced effective beam sizes in normal tissue.

## Conclusion

Interlaced proton minibeams is a spatially fractionated radiation method that substantially spares healthy tissues in comparison to conventional broadbeam irradiation. Interlaced dose distributions from two and four directions were calculated and evaluated with respect to the sparing potential of healthy tissue by comparing mean clonogenic cell survival at a certain distance to the tumor edges. Interlaced minibeam irradiations reveal enhanced mean clonogenic cell survival compared to unidirectional minibeam irradiation. The mean clonogenic cell survival of interlaced proton minibeams profits most, in particular close to the tumor edge, by the application of a heterogeneous tumor dose, in particular taking beams from four directions. The geometrical sparing predominates the reduction of adverse side effects in spite of an increased mean dose, which is required to fill the cold spots within the tumor volume. Thus, side effects are reduced and even higher mean doses are applied to the tumor.

Proton minibeams are of significant advantage for high dose fractions (≥ 10 Gy), i.e. hypofractionation. Hence, single fraction tumor treatment at acceptable side effects even close to the tumor edges comes into reach when applying interlaced minibeams with heterogeneous tumor dose. High cost reductions are expected in spite of the higher effort to produce and adjust the interlaced minibeams.

A main obstacle in using proton minibeams for interlaced irradiation modes is the biologically effective beam size of single minibeams inside the healthy tissue. Especially at larger depths, the lateral spread through multiple small angle scattering limits smaller beam sizes. If needed, smaller effective beam sizes can be achieved using heavy ion minibeams, e.g. using helium or, with pros and cons, carbon ions.

Our findings may pave the way for technical solutions in preclinical and clinical proton or heavy ion minibeam therapy. Altogether, we recommend focusing on high-dose planar minibeams in future interlacing studies due to easier feasibility as well as higher flexibility of irradiation modes. However, pencil minibeams have a two-dimensional repair geometry compared to the one-dimensional one of planar minibeams. Thus, their healthy tissue sparing potential has to be compared at the same or even lower mean cell survival.

## Supplementary Information

Supplementary Information
