# Supplementary material for: Optimizing proton minibeam radiotherapy by interlacing and heterogeneous tumor dose on the basis of calculated clonogenic cell survival
Source: Sci Rep. 2021 Feb 11;11:3533. doi: 10.1038/s41598-021-81708-4 (PMC7878903; doi:10.1038/s41598-021-81708-4)
Supplement: Supplementary file 1 — Supplementary Information [file 41598_2021_81708_MOESM1_ESM.docx]

**Supplementary Material**

*Interlacing from four directions: Comparison to previous studies*

Dilmanian et al. [8] and Henry et al. [25,27] had similar but in detail different approaches when it comes to interlacing from four directions. Their concepts are schematically presented in Figure S1 to be compared with our interlacing approach presented in Figure 1f.


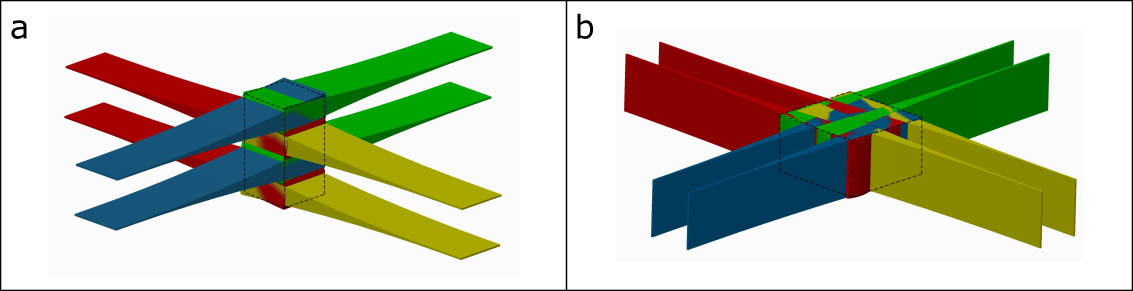


Figure S1. Schematic illustration of 4-dir interlacing as suggested by Dilmanian et al. (a) and Henry et al. (b). Figures are created using the Creo 2.0 software. Credit: Peter Hartung.

Dilmanian et al. suggested a superposed application of planar beams from opposite directions. That means that these beams are not interlaced but meet the same plane within the tumor. The orthogonal directions are interlaced only. This concept leads to lower ctc spacings compared to the approach of Figure 1f where the irradiated planes of all directions are interlaced. Thus, less tissue sparing is expected using this approach compared to the actual 4-dir interlacing (Figure 1f)

Henry et al. suggested the interlacing from opposite directions as suggested for the 2-dir case (Figure 1d) and superposing the orthogonal directions setting the planes perpendicularly to each other. In this case too, the tissue sparing does only profit from an interlacing approach from two directions but does not fully take the advantage when irradiating with minibeams from four directions.

Thus, both approaches are basically interlaced from two directions, but split the dose into four directions. The split-up of the dose as used in conventional radiotherapy plus the strong spatial fractionation leads to an enhanced dose tolerance of the traversed tissues but can keep the tumor dose homogeneous [21,22]. A comparison between the interlacing methods of Dilmanian, Henry and this study on the basis of cell survival are presented in Figure S2a for homogeneous tumor irradiation.


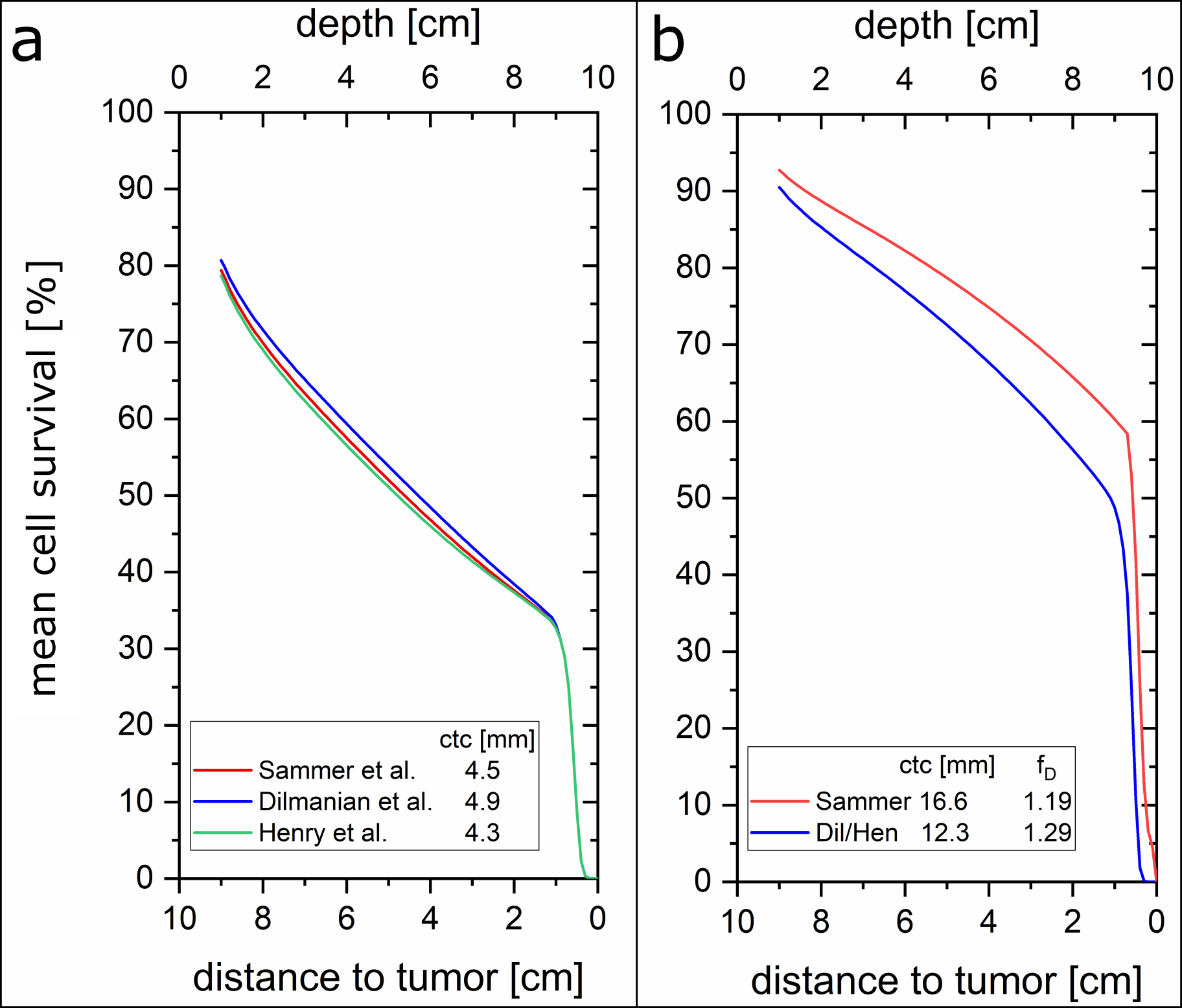


Figure S2. Cell survival of different 4-dir interlacing methods of this study (red line), Dilmanian et al. (blue line) and Henry et al. (green line) for a 10 Gy homogeneous tumor irradiation. The tumor is located in 10 – 15 cm depth.

The differences for homogeneous tumor irradiation are very small between the three arrangements. The scattering within the tumor leads to a slightly enhanced uniformity of Dilmanian’s concept leading to a ctc of 4.9 mm. The interlacing of Henry et al. only allows the same ctc distances as a standard 2-dir interlacing. There is no significant difference between the three methods.

When it comes to heterogeneous irradiation, both approaches of Dilmanian and Henry are similar as 2-dir interlacing with planar beams. The main advantage is based on the dose split-up. A comparison between the 4-dir irradiation concepts of Dilmanian et al. and Henry et al. (Figure S1) and the present approach of Sammer et al. (Figure 1f) with heterogeneous tumor dose (10 Gy minimum) are presented in Figure S2b. Cell survival is larger for the Sammer concept of four interlaced planar beams as suggested in the presented study when comparing to the superposed 2-dir concepts. This enhanced cell survival is already obtained at a lower dose enhancement factor. However, the main advantage is found in the dose enhancement, which needs to be only increased by a factor of f_D_=1.19 rather than f_D_=1.29 in the optimum case (local minimum). Therefore, larger ctcs’ are enabled at lower dose enhancement factors and dose fluctuations in the tumor leading to higher tissue sparing capabilities of healthy tissues.

*Mean dose multiplication factor f_D_*

In the normal tissue, the stronger the dose modulation, the more sparing can be achieved [13]. However, if every cell within the tumor volume should be irradiated with a minimum amount of dose, the mean dose needs to be increased for heterogeneous dose distributions to fulfill this minimum criterion. The stronger the dose heterogeneity the higher the mean dose has to be set to lift the minimal dose D_min_. A schematic illustration of the different dose modulations is shown in Figure S3b/c. Figure S3d displays the same dose modulation as in Figure S3c but with the mean dose increased by a factor f_D_ of 1.5, leading to a lifted minimum dose to approach the required prescribed dose D_t._


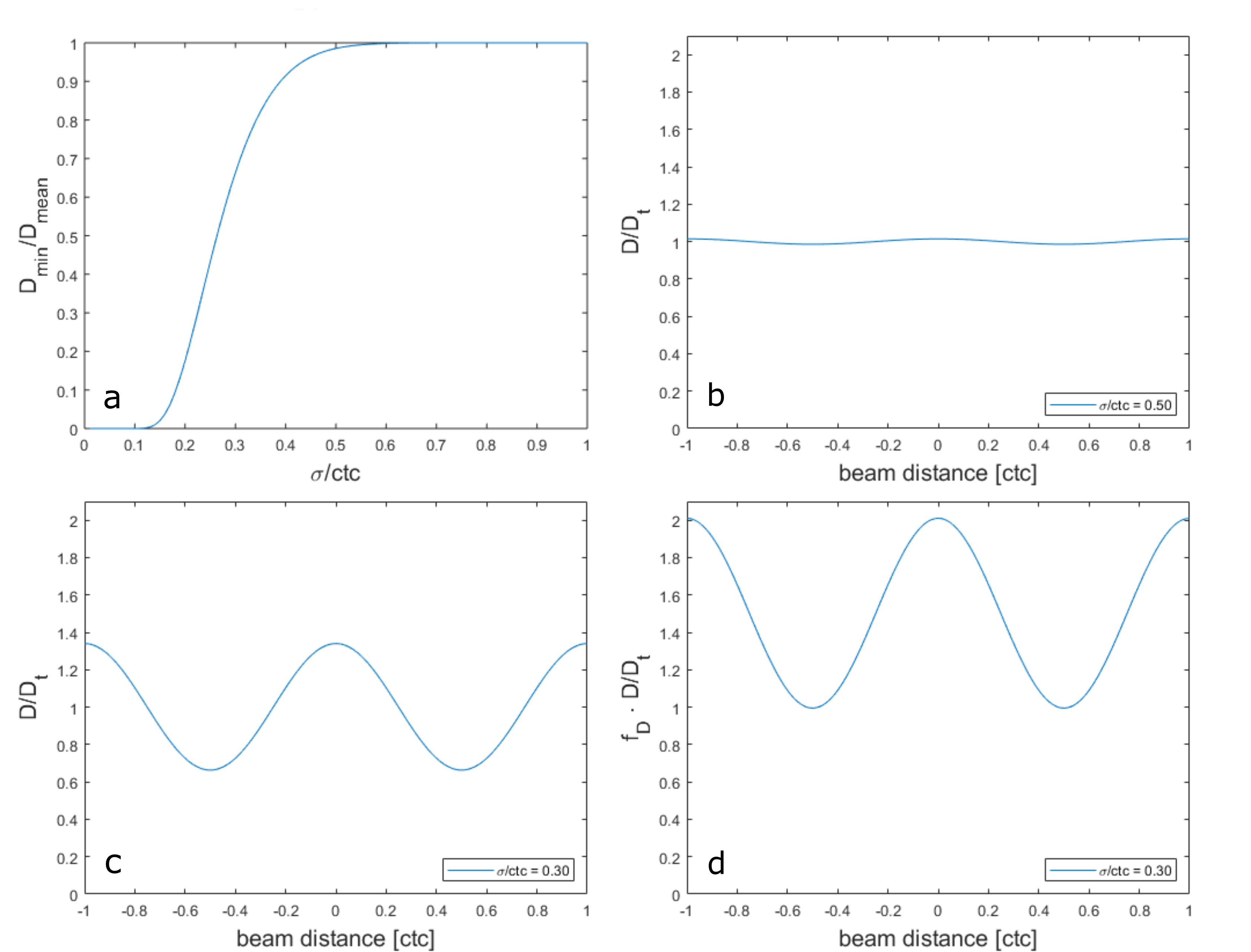


Figure S3. Representative plots for the minimum dose dependency on σ/ctc (a), different dose modulations of σ/ctc = 0.5 (b) and σ/ctc = 0.3 (c) as well as a high dose modulation σ/ctc = 0.3 with increased mean dose by f_D_= 1.5 (d).

The multiplication factor f_D_ in dependence of the ctc distance is displayed in Figure S4a for the 2-dir planar pMBRT irradiation using the 1-Gaussian approach as it is sufficient for the description of the tumor dose modulation. In most of the displayed ctc range, f_D_ increases with ctc, but there is a local minimum due to geometrical effects that occur in interlacing scenarios from two or more directions where in and outgoing beams are of different size (σ_prox_= 1.88 mm and σ_dist_ = 3.17 mm). For a ctc of 7 mm, the distal beam shows almost no dose modulation (red line, Figure S4b) and the overall dose modulation (yellow line) is only determined by the incoming beam (blue line). The proximal dose modulation causes an increase of the mean dose by f_D_=1.24 to fulfill the minimum criterion. In the case of ctc = 9.4 mm (Figure S4c), the valley dose of the incoming beam (blue line) drops more than the maximum of the outgoing beam increases because of the beam size differences. This asymmetric behavior causes the necessity of a local f_D_ maximum at 9.4 mm ctc to lift the minimum dose of the dose sums (yellow line) to 0.975*D_t_. Increasing the ctc to even 12.3 mm (Figure S4d) delivers the, from a dose point of view, ideal situation, where the dose valley of the incoming beam (blue line) and the dose peak of the outgoing beam (red line) compensate each other. The integral dose valley in the yellow line is here the broadest. Increasing the ctc distance even further to 18.8 mm results in the formation of two local minima. The outgoing beam (red line) is too small to cover the full valley of the incoming beam (blue line), which results in the two minima of the steep slopes of neighboring Gaussians. The minima therefore decrease exponentially which is observed in the exponential increase of f_D_ for ctc > 12.3 mm in Figure S4a. The behavior of f_D_ in dependence of ctc depends on irradiation modality (pencil vs. planar and also 2-dir vs. 4-dir) and especially on the in- and outgoing beam sizes which vary with tumor size and location.


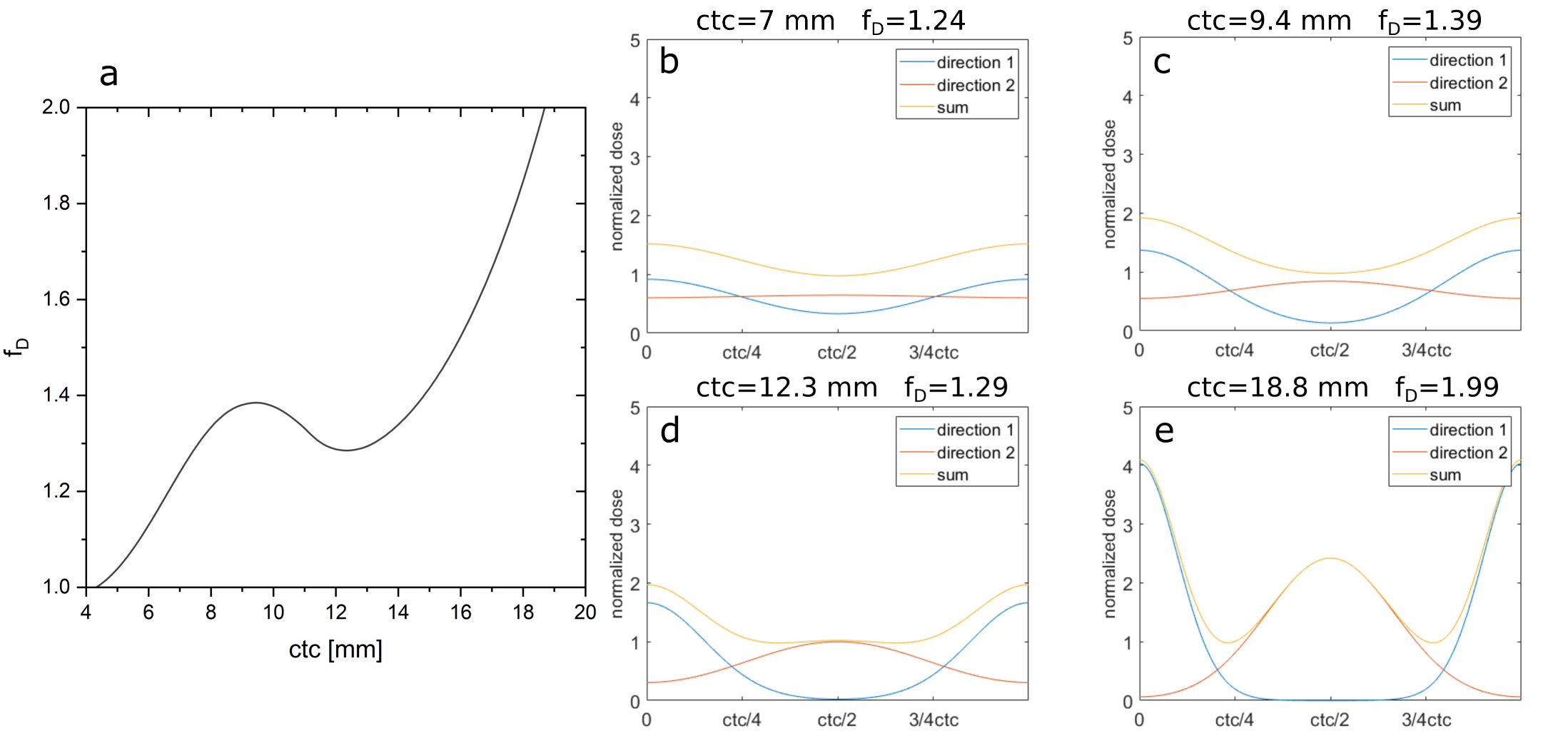


Figure S4. Multiplication factor f_D_ of the mean dose as a function of the ctc distance required to fulfill the minimum criterion D_min_/D_t_ = 0.975 (a). The local dose distributions for different ctc at the proximal tumor depth of 10 cm for different ctc are shown in the subfigures b-e. The shown curves are only valid for the 2-dir planar pMBRT irradiation with the 1-Gaussian approximation.

*Effective beam size*

The effective beam diameter d_eff_ as a function of the maximum dose D_max_ of the Gaussian is displayed in Figure S5 for 1, 10, 50, 90 and 99 % cell survival. It can analytically be described by

$$d_{\mathrm{eff}}\left( \sigma\right)=\frac{2r}{\sigma}=\sqrt{8\ln\left( \frac{2\beta D_{\max}}{-\alpha+\sqrt{\alpha^{2}-4\beta\ln(S)}} \right)},$$

where α and β are the parameter of the LQ-model and S is the survival fraction (0 < S < 1).

The effective beam size is particularly sensitive for changes in the low dose range (~ 0 – 10 Gy) due to the sensitivity of the linear-quadratic model and flattens for high peak doses. Therefore, high peak doses as they appear in the superficial layers of pMBRT cause only a minor increase in the effective beam size, especially considering the small physical beam sizes of only few 100 µm. For instance, an increase of the maximum dose from 100 Gy to 1000 Gy only increases the effective beam diameter by 1-1.5 standard deviations, depending on the cell survival rate. However, at the close tumor surrounding where physical beam sizes are already in the mm range, an increase of the effective beam size of only one standard deviation could decrease the sparing effect of spatial fractionation. The beam sizes where no reaction occurred was determined in a mouse ear model and found to be in the low mm range. A translation into human tissue is necessary, but expectations are of similar dimensions. As already written in the main manuscript, this could be reduced by considering heavier ions as He or C to reduce the lateral scattering of single beams and therefore the absolute value of the effective beam size.


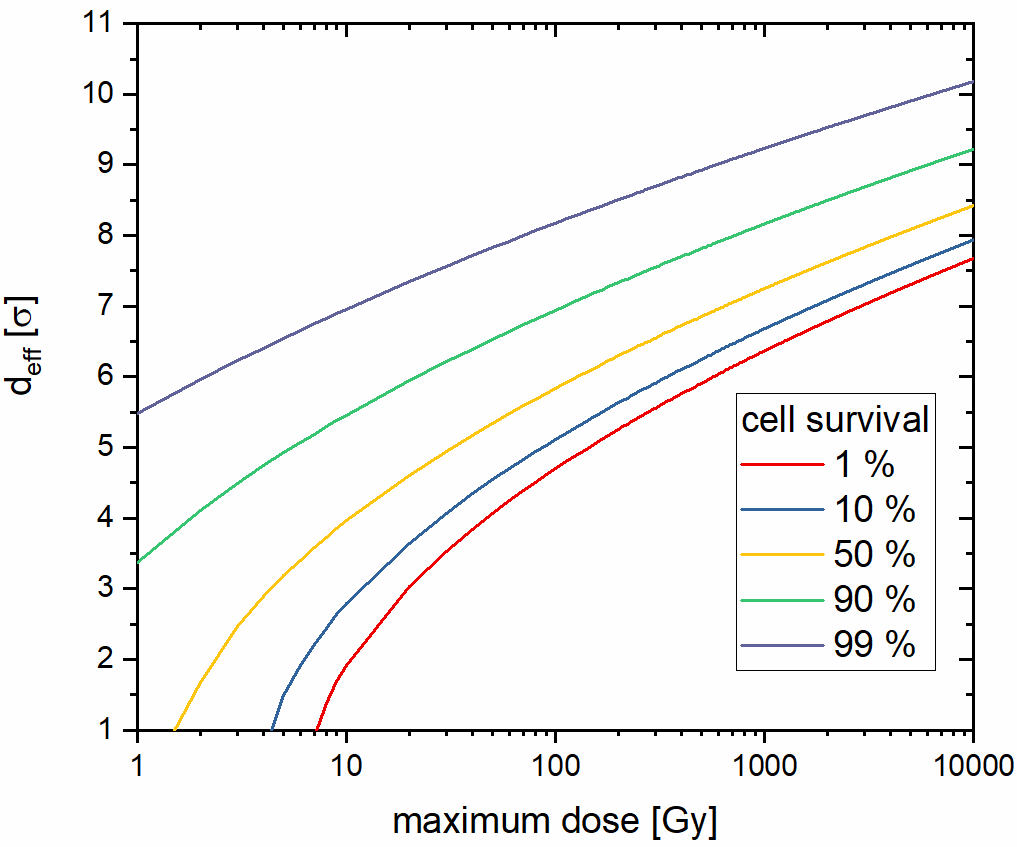


Figure S5. Effective beam size (diameter in number of standard deviations σ) as a function of the maximum dose of a Gaussian dose distribution for five different cell survival values. The α and β values are the utilized α and β values α = 0.425 Gy^-1^ and β = 0.048 Gy^-2^ as in all simulations. Note that the maximum dose axis is displayed on a logarithmic scale.
